# Supplementary material for: MetaRibo-Seq measures translation in microbiomes
Source: Nat Commun. 2020 Jun 29;11:3268. doi: 10.1038/s41467-020-17081-z (PMC7324362; doi:10.1038/s41467-020-17081-z)
Supplement: Supplementary file 10 — Supplementary Data 7 [file 41467_2020_17081_MOESM10_ESM.zip › File2/Confidence_VeryHigh_Taxonomy/181202_out.krona.html]

Javascript must be enabled to view this page.

members
magnitude
magnitudeUnassigned
count
unassigned
taxon
rank

181202\_out

10

2
superkingdom
10

phylum
1239
10

class
186801
10

186802
order
10

10
541000
family

10
genus
1263

1638786
species

SRS014979\_contig\_number\_19489SRS104311\_contig\_number\_39889SRS144183\_contig\_number\_contig-100\_4270.201266
3

6

SRS015431\_contig\_number\_85849SRS019068\_contig\_number\_contig-100\_1230.620264SRS020869\_contig\_number\_23484SRS058070\_contig\_number\_23530SRS104084\_contig\_number\_5321SRS146812\_contig\_number\_contig-100\_1866.278112
species
40519

species
41978
1

SRS078176\_contig\_number\_4156
